# Combination of Markers, Including hsCRP, TGFβ 1, IL-6, and T-regs, and their Predictive Value as Early Warning Signs for Preeclampsia: Insights from a Tertiary Care Medical Centre in Eastern India

**DOI:** 10.34763/jmotherandchild.20263001.d-25-00031

**Published:** 2026-03-15

**Authors:** Prakruti Dash, Bharath Kumar Koppisetty, Saubhagya Kumar Jena, Saurav Nayak

**Affiliations:** Additional Professor, Department of Biochemistry, All India Institute of Medical Sciences, Bhubaneswar, Odisha, India; Senior Resident, Department of Biochemistry, All India Institute of Medical Sciences, Mangalagiri, Andhra Pradesh, India; Professor and Head, Department of Obstetrics and Gynecology, All India Institute of Medical Sciences, Bhubaneswar, Odisha, India; Assistant Professor, Department of Biochemistry, IMS and SUM Hospital Campus 2, Bhubaneswar, Odisha, India

**Keywords:** preeclampsia, normal pregnancy, Regulatory T cells (T-regs), inflammatory cytokines, foeto maternal immunity

## Abstract

**Background:**

Regulatory T cells (T-regs), a subset of CD4+ T-lymphocytes play a crucial role in the maintenance of immunological tolerance and immune homeostasis. The study has been done to evaluate the threshold values and reference intervals of Regulatory T cells, TGF-β1, IL-6, and hsCRP as a comprehensive panel for preeclampsia.

**Materials and Methods:**

In this cross-sectional study the T-regulatory cells (CD4, CD25, FOXP3) were assessed using a flow-cytometer, and TGF-β1, and IL-6 were analysed by sandwich ELISA method and hsCRP analysed by Beckman Coulter autoanalyser.

**Statistical Analysis:**

The reference range for the parameters in the studied groups was estimated based on CLSI C28-A3 guidelines. The lower and upper limits were estimated by a non-parametric method with 90% CI. Threshold values were estimated from the ROC curve based on optimal sensitivity and specificity measured by the Youden Index.

**Results:**

The study established a threshold value level for CD3+CD4+T-cells, CD4+CD25+T-regs, and CD4+FOXP3+ T-regs to separate individuals with preeclampsia from those without the condition. The threshold values for TGF-β1, IL-6, and hsCRP were chosen as they demonstrated significant differences between the two groups, indicating an association with the presence of preeclampsia.

**Conclusion:**

The statistical analysis of the study population aimed to establish a clear difference between the two groups, thereby providing a dependable foundation to differentiate impending preeclampsia from a normal pregnancy in order to assist in theranostics.

## Introduction

The diagnosis of preeclampsia, a complicated multisystem illness, is made up of at least one significant correlated issue, such as proteinuria, maternal organ malfunction, or uteroplacental dysfunction, along with rapid onset hypertension (>20 weeks of gestation) [1]. One of the most severe pregnancy disorders, preeclampsia is a major cause of morbidity and mortality for both mothers and children. Preeclampsia affects an estimated 4 million women globally. In healthcare facilities in India, preeclampsia rates range from 5% to 15% [2].

Preeclampsia’s aetiology continues to remain unknown, although a growing body of research points to immune system involvement. An impaired tolerance to the conceptus is a key component of the pathogenesis, especially in cases of early-onset and severe preeclampsia [3]. The ultimate consequence is heightened systemic inflammation accompanied by circulating T cell activation and elevated interferon-gamma (IFN-γ) production. Thus, it makes sense to believe that preeclampsia may arise as a result of a malfunction in the control of T cells [3,4]. One of the primary immune regulators is Regulatory T (T-reg) cells. They suppress an array of cell types, including T cells, through methods that remain incompletely understood but include cell-cell contact and the release of soluble mediators like Transforming Growth Factor (TGF-beta). Forkhead-Box P3 (FOXP3)-expressing CD4+ T helper cells are considered to be the primary Regulatory T cells in humans [5,6].

There is mounting evidence that TGF-β1 is crucial for immune-mediated homeostasis, immune cell differentiation regulation, and immune cell function maintenance. Maintaining foetal-maternal immune tolerance requires careful coordination of the actions of different immune cells and cytokines [7]. TGF-β1 is crucial for trophoblast cell invasion, foetal-maternal immune tolerance maintenance, and remodelling of the uterine spiral artery. Furthermore, TGF-β1 is an adaptive cytokine that regulates immune cell function extensively and is essential for foetal-maternal immunological tolerance thus playing a pivotal role in preeclampsia [8,9].

Both local and systemic responses to inflammation arise from the activation of pro-inflammatory cytokines, as observed in pregnant women with preeclampsia. Pro-inflammatory cytokine levels in the serum, such as IL-6 (Interleukin – 6), are also much higher in women with preeclampsia [10]. This state of inflammation also keeps steadily increasing levels of markers of systemic inflammation high in these pregnant females [11]. Given the significance of these factors in the development of preeclampsia, our research intends to establish threshold values and reference ranges for T-regs, IL6, TGF-β1, and High-Sensitive C-Reactive Protein (hsCRP) as a complete panel for preeclampsia, with the goal of distinguishing it from levels observed in normal pregnancies.

## Materials and Methods

The study was conducted in the Department of Biochemistry in conjunction with the Department of Obstetrics and Gynecology at All India Institute of Medical Sciences (AIIMS), Bhubaneswar. Prior approval of the Institutional Ethical Committee was taken vide *IEC/AIIMS BBSR/PG Thesis/2021-22/40*. Written informed consent was acquired from all participants voluntarily.

In the cross-sectional study 40 participants were recruited, with 20 cases of preeclampsia and 20 normal pregnant females. All the cases were of early onset preeclampsia, selected at the time of first diagnosis and before 34 weeks of gestation. After getting consent, 5 ml venous blood was collected from each study subject. A 2 ml blood sample was collected in an EDTA (Ethylenediaminetetraacetic acid) vacutainer tube for the estimation of Regulatory T cells (CD4, CD25, and FOXP3) by flow cytometry. The remaining amount was collected in a plain vacutainer tube. Serum was separated and stored at −20 degrees Celsius and was used for the estimation of IL-6, TGF-β1, and hsCRP. DURAClone® IM T-regs tubes by Beckman Coulter were used for flow cytometry analysis of Regulatory T cells. The DURAClone® IM T-reg panel enables the identification and characterisation of Regulatory T cells by using a fast permeabilisation protocol. Sandwich ELISA (Enzyme Linked Immunosorbent Assay) was used for IL-6 and TGF-β1. A Beckman Coulter 5800 Autoanalyser was used for hsCRP analysis. The absolute count was obtained by multiplying the T-regs % value with the corresponding appropriate lymphocyte count.

### Statistical Analysis

All analysis will be done using IBM SPSS v26.0 and Python 3.6. The reference range for the parameters in the studied groups was estimated based on CLSI C28-A3 guidelines. The lower and upper limits were estimated by a non-parametric method with 90% CI (Confidence Interval). Threshold values were estimated from the ROC (Receiver Operating Characteristic) curve based on optimal sensitivity and specificity measured by the Youden Index and Area Under Curve (AUC). A *p*-value of <0.05 was considered to be statistically significant.

## Results

In the study population it was seen that the level of CD4+FOXP3+ cells, as well as IL-6 and hsCRP levels is significantly increased in preeclampsia patients,. The demographic, anthropometric and immunological data of the study population has been tabulated [Table 1].

**Table 1.** Descriptive statistics of group-wise data and comparison by Mann-Whitney *U* test.

**Parameters**	**Normal Pregnant Women**	**Pregnant with Preeclampsia**	***p*-value (Mann-Whitney U)**
Age (in years)	28 (25 – 30)	28 (23 – 30)	0.989
Gestational Age (in weeks)	36.3 (28.85 – 38.35)	36.9 (33.8 – 38.15)	0.698
Weight (in kg)	55.5 (51 – 62.5)	54.5 (48 – 59.5)	0.429
Height (in cms)	153.5 (149 – 158)	152.5 (148 – 158)	0.495
BMI (in kg/m^2^)	24.365 (22.87 – 25.175)	24.12 (21.68 – 26.505)	0.947
Waist To Hip Ratio	0.85 (0.82 – 0.865)	0.845 (0.82 – 0.85)	0.547
Systolic BP (in mm of Hg)	120 (110 – 120)	147 (140 – 159.5)	**<0.001**
Diastolic BP (in mm of Hg)	79 (70 – 80)	100 (97 – 110)	**<0.001**
Lymphocyte %	8.28 (6.14 – 11.72)	10.695 (8.57 – 13.9)	0.159
Absolute Lymphocytes (in no. of cells)	8280 (6140 – 11720)	10695 (8570 – 13900)	0.159
CD3+CD4+ %	33.93 (29.67 – 36.91)	30.26 (27.12 – 39.6)	0.374
CD4+CD25+ %	5.17 (2.64 – 7.16)	3.495 (1.9 – 5.8)	0.159
CD4+FOXP3+ %	4.82 (4.22 – 5.46)	1.615 (1.17 – 1.94)	**<0.001**
Absolute CD3+CD4+ (in no. of cells)	11322 (9901 – 12317)	10097.5 (9050 – 13215)	0.374
Absolute CD4+CD25+ (in no. of cells))	1725 (881 – 2389)	1166.5 (634 – 1935)	0.159
Absolute CD4+FOXP3+ (in no. of cells)	1608 (1408 – 1822)	539 (390 – 647)	**<0.001**
TGF-β1 (in pg/ml)	6229.245 (3023.31 – 6762.24)	6445.635 (5275.9 – 6666.03)	0.718
IL-6 (in pg/ml)	12.405 (9.95 – 15.8)	35.195 (22.37 – 49.92)	**<0.001**
hsCRP (in mg/ml)	3.245 (2.205 – 5.06)	6.965 (3.355 – 28.675)	**0.011**

TGFβ 1-Transforming Growth Factor Beta 1.

BMI-Body Mass Index.

hsCRP- High Sensitive C-Reactive Protein.

In Table 1, descriptive statistics of the group-wise data and comparisons made using the Mann-Whitney *U* test are presented. As anticipated, there was a statistically significant difference in both systolic and diastolic blood pressure between the control group and the cases. The specific parameters examined, namely Regulatory T cells, IL-6, and hsCRP, exhibited significant differences between cases and controls, with T-regs showing a notable decrease and IL-6 and hsCRP showing a significant increase in preeclampsia cases when compared to normal pregnant controls.

**Figure:**
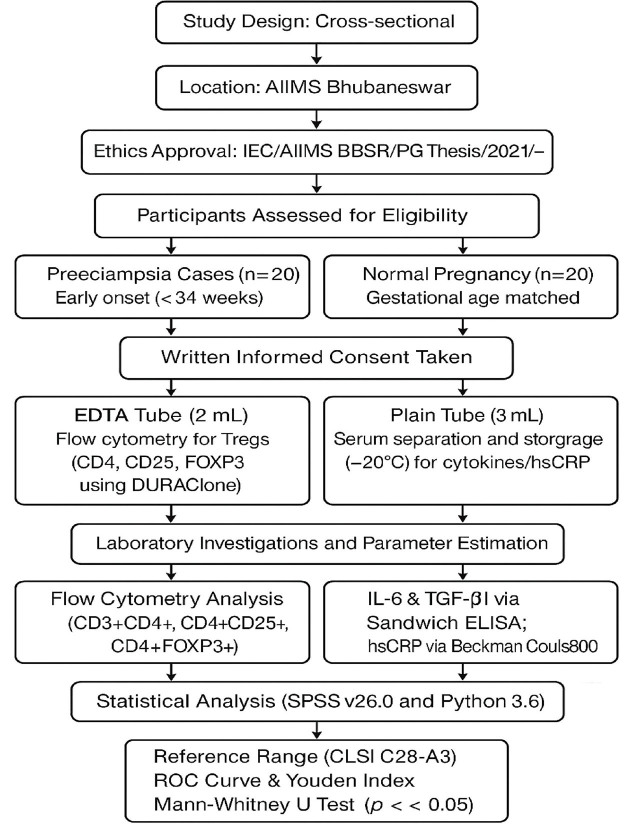
A Flow diagram illustrating the workflow of the study

**Figure 1.**
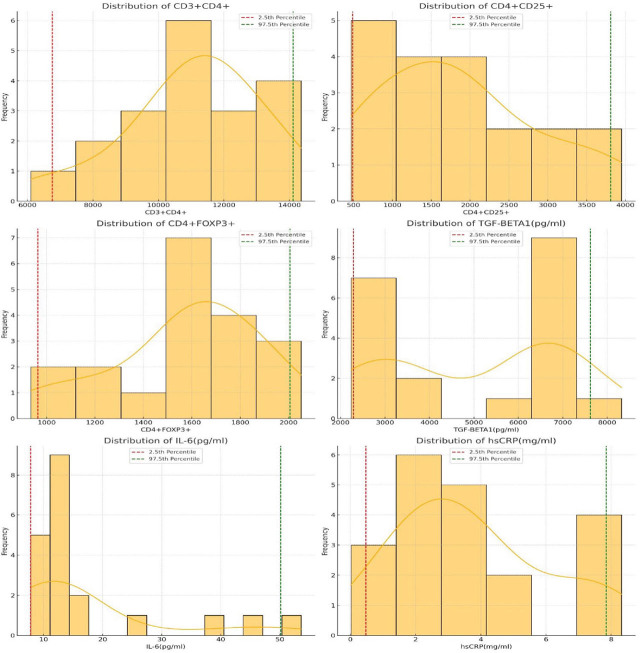
Reference range determination, the normal reference ranges of the studied parameters.

By the robust method of reference range determination, the normal reference ranges of the studied parameters were estimated. The values have been tabulated [Table 2 and Figure 1].

**Table 2.** Lower and Upper Limit of Reference Range by Robust Method with 90%CI.

**Parameter**	**Lower Limit (90% CI)**	**Upper Limit (90% CI)**	**Overall Reference Range**
CD3+CD4+ (no. of cells)	6758.95 (6097 – 6097)	14109.7 (13805 – 14359)	6758.95 – 14109.7
CD4+CD25+ (no. of cells)	486.8 (467 – 467)	3809.7 (3637 – 3951)	486.8 – 3809.7
CD4+FOXP3+ (no. of cells)	964.15 (934 – 934)	2005.65 (1949 – 2052)	964.15 – 2005.65
TGF-β1 (pg/ml)	2290.35875 (2234.76 – 2234.76)	7619.36325 (6843.45 – 8321.38)	2290.35875 – 7619.36325
IL-6 (pg/ml)	7.82 (7.82 – 7.82)	50.12775 (46.29 – 53.6)	7.82 – 50.12775
hsCRP (mg/ml)	0.47125 (0.02 – 0.02)	7.845 (7.32 – 8.32)	0.47125 – 7.845

TGFβ 1-Transforming Growth Factor Beta 1.

IL-6 – Interleukin 6.

hsCRP - hsCRP- High Sensitive C-Reactive Protein.

CI-Confidence Interval.

The total count of CD3+CD4+ cells ranged from a lower limit of 6758.95 to an upper limit of 14109.7. The CD4+CD25+ cells exhibited a lower limit of 486.8 and an upper limit of 3809.7. For CD4+FOXP3+ cells, the lower limit was 964.15, while the upper limit reached 2005.65. TGF-β1 (pg/ml) presented lower and upper limit values of 2290.35875 and 7619.36325, respectively, with IL-6 (pg/ml) showing a range of 7.82 – 50.12775. The reference range for hsCRP (mg/ml) was between 0.47125 and 7.845.

**Figure 2.**
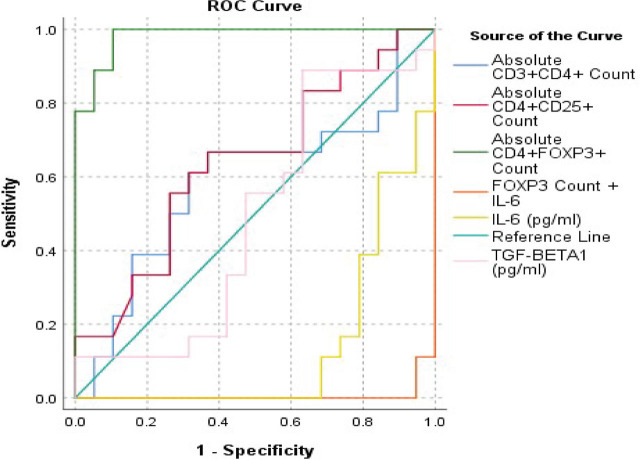
ROC Curve analysis for evaluation of diagnostic efficacy of the studied parameters.

A ROC Curve analysis was performed to evaluate the diagnostic efficacy of the studied parameters. FOXP3 count and IL-6 levels were the only significant parameters, with FOXP3 count being the best parameter individually [AUC = 0.982, *p*<0.001]. IL-6 showed potential diagnostic efficacy with AUC 0.863 and CI of 0.743 – 0.98 with *p*-value *p*<0.001. A combination of FOXP3 count with IL-6 level had the highest diagnostic potential [AUC = 0.994, *p*<0.001]. The ROC Curve analysis is shown in Figure 2, and the AUC data is tabulated [Table 3].

**Table 3.** Area Under Curve analysis of the studied parameters.

**Test Result Variable(s)**	**AUC (95% CI)**	***p*-value**
Absolute CD3+CD4+ Count	0.588 (0.434 – 0.792)	0.378
Absolute CD4+CD25+ Count	0.637 (0.437 – 0.781)	0.142
Absolute CD4+FOXP3+ Count	**0.982 (0.950 – 1.0)**	<0.001
TGF-β1 (pg/ml)	0.391 (0.211 – 0.220)	0.931
IL-6 (pg/ml)	**0.863 (0.743 – 0.982)**	**<0.001**
FOXP3 Count + IL-6	**0.994 (0.982 – 1.008)**	**<0.001**

TGFβ 1-Transforming Growth Factor Beta 1.

IL-6 – Interleukin 6.

With a threshold value of ≤10305 absolute count, CD3+CD4+ showed a sensitivity of 61.11% and specificity of 75% and with an absolute count of ≤1184, CD4+CD25+ showed a 55.56% sensitivity and 67.86% specificity for preeclampsia. CD4+FOXP3+ revealed a 100% specificity and 89.29% specificity with an absolute count of ≤1098. Amongst the inflammatory markers IL-6 with a cut off value of >18.66 pg/ml revealed an 85% sensitivity and 76.67% specificity compared to hsCRP which showed an 80% sensitivity and 66.67% specificity with a cut of value of >3.01 mg/ml. TGF-β1 showed 85% sensitivity and 53.33 % specificity at a cut off of >4175.33 pg/ml [Table 4]. A combination of IL-6 and CD4+FOXP3+ showed the highest diagnostic potential to differentiate between preeclampsia and normal pregnancy as revealed by the ROC curve analysis [Figure 2, Table 3].

**Table 4.** Threshold value of the studied immunological parameters.

**Parameter**	**Cut-Off Value**	**Sensitivity**	**Specificity**
Absolute CD3+CD4+ count	≤10305	61.11	75.00
Absolute CD4+CD25+ count	≤1184	55.56	67.86
Absolute CD4+FOXP3+ count	≤1098	100.00	89.29
TGF-β1 (pg/ml)	>4175.33	85.00	53.33
IL-6 (pg/ml)	>18.66	85.00	76.67
hsCRP (mg/ml)	>3.01	80.00	66.67

TGFβ 1-Transforming Growth Factor Beta 1.

IL-6 – Interleukin 6.

hsCRP - High Sensitive C-Reactive Protein.

## Discussion

Preeclampsia continues to be one of the most serious complications of pregnancy with a multifactorial causation making early prediction difficult. Therefore, our study as its primary objective aimed at providing a clear immunological criterion based on T-regs for early diagnosis of preeclampsia in pregnant females. There existed an ambivalence of T-regs frequency in preeclampsia. Early studies showed that there was an expansion of T-regs subset in normal pregnancy [12,13]. However, this idea has subsequently been countered by further studies, showing non-significant increase in the levels [14,15]. In contention to both, our study found a marked decrease in T-regs level in pregnant females with preeclampsia.

CD4+ T-reg cells are crucial for early placental development and embryo implantation, among other mechanisms of maternal tolerance. Throughout gestation, their ability to limit and alleviate the inflammation caused by embryo implantation and inhibit the production of immune effector cells in nearby lymph nodes is essential for regulating inflammation and maintaining immunological tolerance [16,17]. All but two of the 17 individual principal studies that were examined in a recent meta-analysis provided consistent evidence of a link between reduced T-reg cells in the third trimester and severe, early-onset, and late-onset preeclampsia [18]. In addition to their decreased number, T-reg cells’ ability to inhibit severe immune response is frequently impaired in preeclampsia. The severity of the disease may be correlated with the decline in T-reg cells [19]. Our research revealed a notable reduction in T-regs in pregnant women experiencing preeclampsia compared to those with normal pregnancies, demonstrating considerable sensitivity and specificity in distinguishing preeclampsia from normal pregnancy based on the determined threshold values.

Research has shown that immunological cells, epithelial cells, and placental trophoblasts and decidual cells all release IL-6 [10]. Numerous inflammatory cytokines are linked to several pathophysiological events of pregnancy, including maternal infection, placental ischaemia, and decreased uterine blood flow. This inflammatory reaction could be a factor in preeclampsia [20,21,22]. The significant difference in IL-6 levels between preeclampsia and normal pregnancies suggests it could serve as a diagnostic marker for preeclampsia cases. However, it is worth noting that it is a non-specific inflammatory cytokine. Additionally, since our study is cross-sectional, a prospective cohort study is necessary to track IL-6 changes throughout pregnancy and establish a threshold value for the progression from gestational hypertension to overt preeclampsia. When used alongside T-regs, the precision of these tests nearly reaches an impeccable standard, demonstrated by a significantly high AUC for differentiating preeclampsia from normal pregnancies. As mentioned above future cohort studies can further investigate the combination of markers such as T-regs, IL-6, TGFβ 1, and hsCRP to validate their diagnostic effectiveness as early indicators for the onset of preeclampsia in cases of pregnancy-induced hypertension (PIH).

Our research was one of the key studies conducted in India, particularly in Eastern India, where the incidence of preeclampsia is increasing and often remains undetected until later stages. In this study, an effort was made to establish a threshold value for T-regs and inflammatory cytokines to accurately distinguish between preeclampsia and normal pregnancy. In order to utilise the panel of parameters analysed and assess its effectiveness in diagnosing preeclampsia at an early stage prior to the appearance of proteinuria, a prospective cohort methodology is necessary.

## Limitations

There were very few diagnosed cases of preeclampsia attending our tertiary care setup thus reducing our sample size. A multicentric study with a larger scope will be an effective way forward to provide effective diagnostic capability to pregnant mothers. The instances of preeclampsia characterised by elevated inflammatory cytokines and decreased T-regs were not monitored for disease progression. The current research did not assess maternal and foetal outcomes. These findings could enhance the overall significance of the study’s results.

Additionally, this study was cross-sectional in nature. The threshold values identified may be useful for distinguishing early onset preeclampsia from normal pregnancy. To assess the set of parameters for the early identification of preeclampsia or the advancement of pregnancy-induced hypertension to preeclampsia, a prospective cohort study with follow-up is required.

This information can be viewed as preliminary research that may offer important insights for future projects concerning the establishment of threshold values for the parameters examined in preeclampsia.

## Conclusion

Regulatory T cells are crucial in modulating the immune response during pregnancy, highlighting their significant theranostic potential in complicated situations like preeclampsia. When paired with cytokines such as IL-6, their ability to differentiate between preeclampsia and normal pregnancy improves, enhancing their clinical efficacy. This combination serves as a powerful tool for the management of pregnancies at risk of developing preeclampsia throughout gestation.

### Key Points

➢ Regulatory T cells are crucial in modulating the immune response during pregnancy.
➢ A reduced quantity of T-reg cells can lead to an improper immune response thus playing a role in the development of preeclampsia.
➢ The severity of the disease may be correlated with the decline in T-reg cells.
➢ Local and systemic responses to inflammation arise from the activation of pro-inflammatory cytokines, as observed in pregnant women with preeclampsia.
➢ Combination of markers such as T-regs, IL-6, TGFβ 1, and hsCRP can be used as early indicators for the onset of preeclampsia in cases of pregnancy-induced hypertension.
➢ The established threshold values and reference intervals could assist in the theranostics of preeclampsia.
